# The complete chloroplast genome sequence of *Vitellaria paradoxa*

**DOI:** 10.1080/23802359.2019.1636727

**Published:** 2019-07-12

**Authors:** Yi Wang, Xiaolong Yuan, Zhonghua Chen, Ting Luo

**Affiliations:** Laboratory of Forest Plant Cultivation and Utilization, Yunnan Academy of Forestry, Kunming Yunnan, People's Republic of China

**Keywords:** *Vitellaria paradoxa*, chloroplast, Illumina sequencing, phylogenetic analysis

## Abstract

*Vitellaria paradoxa* is an important economical tree in Africa. In this study, the complete chloroplast genome (cpDNA) sequence of *V. paradoxa* was determined from Illumina HiSeq pair-end sequencing data. The cpDNA is 159,725 bp in length, contains a large single copy region (LSC) of 88,720 bp and a small single copy region (SSC) of 19,338 bp, which were separated by a pair of inverted repeat (IR) regions of 25,981 bp. The genome contains 130 genes, including 85 protein-coding genes, 8 ribosomal RNA genes, and 38 transfer RNA genes. The overall GC content of the whole genome is 36.7% and the corresponding values of the LSC, SSC, and IR regions are 34.6, 30.0, and 42.9%, respectively. Further phylogenomic analysis showed that *V. paradoxa* clustered together with *Sideroxylon wightianum.*

*Vitellaria paradoxa* is an important economical tree in Africa (Maranz et al. 2004). *Vitellaria paradoxa* yields a high-stearate fat, called Shea butter, that is extracted from the large seeds. Shea butter can be used as a valuable product in the food and cosmetic industries (Wei et al. 2019). *Vitellaria paradoxa* was introduced into China from Ghana and planted in Yuanjiang, Yunnan province, in 1964 (Yang and Wang 2018). The Shea nuts and butter of *V. paradoxa* are important export commodities and play a major role in the local economy and diet, the fruit pulp is also widely consumed (Maranz et al. 2003). Recently, the researcher reported that the leaf and bark of *V. paradoxa* showed antimicrobial activities (Olasunkanmi et al. 2017). However, there have been no genomic studies on *V. paradoxa*.

Herein, we reported and characterized the complete *V. paradoxa* plastid genome (MK953548). One *V. paradoxa* individual (specimen number: 2018040131) was collected from Yuanjiang, Yunnan Province of China (23°19′23″ N, 101°30′19″ E). The specimen is stored at Yunnan Academy of Forestry Herbarium. DNA was extracted from its fresh leaves using DNA Plantzol Reagent (Invitrogen, Carlsbad, CA, USA).

Paired-end reads were sequenced by using Illumina HiSeq system (Illumina, San Diego, CA, USA). In total, about 29.9 million high-quality clean reads were generated with adaptors trimmed. Aligning, assembly, and annotation were conducted by CLC de novo assembler (CLC Bio, Aarhus, Denmark), BLAST, GeSeq (Tillich et al. 2017), and GENEIOUS v 11.0.5 (Biomatters Ltd, Auckland, New Zealand). To confirm the phylogenetic position of *V. paradoxa*, other nine species of order Ericales from NCBI were aligned using MAFFT v.7 (Katoh and Standley 2013) and maximum likelihood (ML) bootstrap analysis was conducted using RAxML (Stamatakis 2006); bootstrap probability values were calculated from 1000 replicates. *Cornus capitata* (MG524990) and *Alangium alpinum* (MG525003) were served as the out-group.

The complete *V. paradoxa* plastid genome is a circular DNA molecule with the length of 159,725 bp, contains a large single copy region (LSC) of 88,720 bp and a small single copy region (SSC) of 19,338 bp, which were separated by a pair of inverted repeat (IR) regions of 25,981 bp. The overall GC content of the whole genome is 36.7% and the corresponding values of the LSC, SSC, and IR regions are 34.6, 30.0, and 42.9%, respectively. The genome contains 130 genes, including 85 protein-coding genes, 8 ribosomal RNA genes, and 38 transfer RNA genes. Phylogenetic analysis showed that *V. paradoxa* clustered together with *Sideroxylon wightianum* (Figure 1). The determination of the complete plastid genome sequences provided new molecular data to illuminate the Ericales evolution.

**Figure 1.**
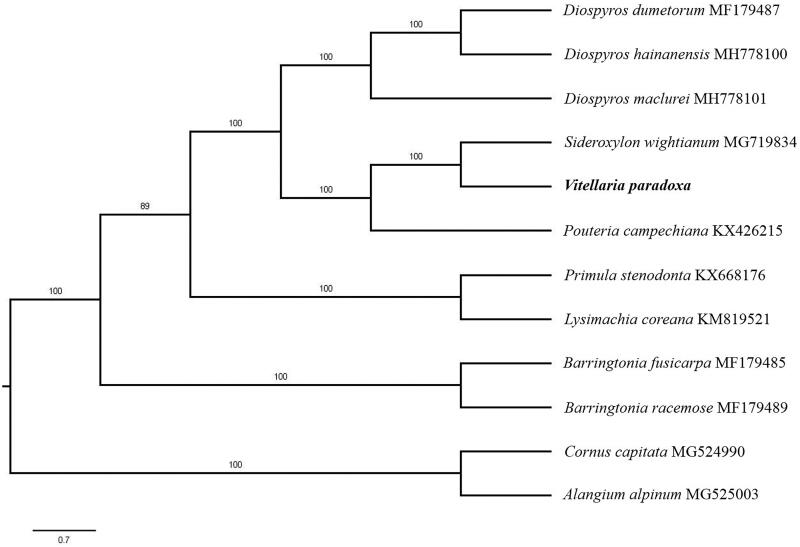
The maximum-likelihood tree based on the 9 chloroplast genomes of order Ericales. The bootstrap value based on 1000 replicates is shown on each node.
